# Potential Therapeutic Benefits of Honey in Neurological Disorders: The Role of Polyphenols

**DOI:** 10.3390/molecules27103297

**Published:** 2022-05-20

**Authors:** Arslan Iftikhar, Rimsha Nausheen, Humaira Muzaffar, Muhammad Ahsan Naeem, Muhammad Farooq, Mohsin Khurshid, Ahmad Almatroudi, Faris Alrumaihi, Khaled S. Allemailem, Haseeb Anwar

**Affiliations:** 1 Department of Physiology, Government College University Faisalabad, Faisalabad 38000, Pakistan; arslaniftikhar@gcuf.edu.pk (A.I.); rimshanosheen41@gmail.com (R.N.); drhumairamuzaffar@gcuf.edu.pk (H.M.); 2 Department of Basic Sciences, KBCMA College of Veterinary and Animal Sciences, Narowal 51600, Pakistan; ahsan.naeem@uvas.edu.pk; 3 Department of Clinical Sciences, College of Veterinary and Animal Sciences, Jhang 35200, Pakistan; muhammad.farooq@uvas.edu.pk; 4 Department of Microbiology, Government College University Faisalabad, Faisalabad 38000, Pakistan; mohsinkhurshid@gcuf.edu.pk; 5 Department of Medical Laboratories, College of Applied Medical Sciences, Qassim University, Buraydah 51452, Saudi Arabia; aamtrody@qu.edu.sa (A.A.); f_alrumaihi@qu.edu.sa (F.A.)

**Keywords:** honey, polyphenols, longevity, flavonoids

## Abstract

Honey is the principal premier product of beekeeping familiar to Homo for centuries. In every geological era and culture, evidence can be traced to the potential usefulness of honey in several ailments. With the advent of recent scientific approaches, honey has been proclaimed as a potent complementary and alternative medicine for the management and treatment of several maladies including various neurological disorders such as Alzheimer’s disease, Parkinson’s disease, Huntington’s disease, and multiple sclerosis, etc. In the literature archive, oxidative stress and the deprivation of antioxidants are believed to be the paramount cause of many of these neuropathies. Since different types of honey are abundant with certain antioxidants, primarily in the form of diverse polyphenols, honey is undoubtedly a strong pharmaceutic candidate against multiple neurological diseases. In this review, we have indexed and comprehended the involved mechanisms of various constituent polyphenols including different phenolic acids, flavonoids, and other phytochemicals that manifest multiple antioxidant effects in various neurological disorders. All these mechanistic interpretations of the nutritious components of honey explain and justify the potential recommendation of sweet nectar in ameliorating the burden of neurological disorders that have significantly increased across the world in the last few decades.

## 1. Introduction

Honey is the primary product of apiculture with a history of use corresponding to the history of mankind. The nutritional and therapeutic benefits of honey have been indicated in every culture and religion of the world including Greek, Roman, Christianity, and Islam [1,2]. Physicians of ancient times have extensively discussed the medicinal qualities of honey. Ancient Egyptian physicians employed honey in their medication 5000 years ago and the prehistoric Greeks believed honey beneficial for vigor and longevity. The first written reference to the beneficial effects of honey, a Sumerian tablet writing, dating back to 2100–2000 B.C., indicates honey as a potential drug to be used against various complaints. Ibn e Sina, also known as Avicenna in the west, employed honey as a remedy for tuberculosis [3,4]. Ancient Egyptian physicians used to prescribe a mixture of honey, grease, and fiber for wound healing. It also held a high rank in Chinese medicine and was used for wound healing and various gut diseases. Hippocrates, the Greek physician also known as the Father of Medicine, endorsed the use of a mixture of honey and water (hydromel) for quenching thirst. He also recommended the combined use of honey and vinegar (oxymel) for relieving pain. Many other eminent personnel including Aristotle, Aristoxenus, Porphyry, Cornelius Celsus, and Dioscorides were also convinced of the beneficial effects of different kinds of honey in various illnesses [5,6].

Honey has also been indicated as a potential remedy for various ailments in almost all prevailing religions. In the bible, the word ‘honey’ has been mentioned about 61 times and there are at least six verses that suggest the beneficial effects of honey (Exodus 3:6–8; Samuel 14:24–27; Genesis 43:11; Proverbs 24:13; Matthew 3:1–4; Revelation 10:7–11). In Islam, both the Quran and Hadiths (Prophetic traditions) refer to honey as a source of healing in various diseases (Al-Quran 16:68–69; Sunan Ibn Majah 32:3578). The prophet Muhammad recommended honey for various purposes, including for the treatment of ailments related to the digestive system (Sahih Muslim 39:5770), heart (Sahih al-Bukhari 76:12), and healing phenomenon (Sahih al-Bukhari 76:4). The Almighty’s divine claim about this wonderful liquid gold not only advocates the use of honey for the prevention and treatment of various disorders, but also instigates the eagerness to explore more scientific rationalization for its unrevealed and mysterious beneficial effects.

## 2. Composition of Honey

The beneficial effects of honey are attributed to various biological bioactive components. The presence of these active compounds justifies the substantial biological benefits of honey. The percentage of all these components varies among different types of honey. In general, honey consists of more than 200 substances. It is mainly a carbohydrate product, and sugars constitute more than 90% of solids. Main sugars found in honey include glucose, sucrose, maltose, fructose, melezitose, isomaltose, maltulose, turanose, nigerose, melibiose, panose, and maltotriose. Water is the second most important component of honey. Conjointly with carbohydrates and water, honey also contains enzymes, vitamins, minerals, flavonoids, and polyphenols [7,8,9]. Riboflavin (Vit. B2), Niacin (Vit. B3), Pantothenic acid (Vit. B5), Pyridoxine (Vit. B6), Folate (Vit. B9), and vitamin C are the major vitamins found in honey. Among minerals, potassium is the major one while calcium, magnesium, sodium, sulfur, and phosphorus are also found in a significant amount. The main enzymes found in honey include invertase (saccharase), diastase (amylase), and glucose oxidase. Non-enzymatic proteins, including glycoprotein, MRJP1, and apalbumin-1, are also found in honey, but in very minute quantities [9].

Polyphenols are a major class of naturally occurring organic compounds that are defined by multiples of phenol units. Polyphenols consist of flavonoids and nonflavonoids that are separated into subclasses based on the number of phenol units, substituent groups, and/or the kind of linkage between phenol units in their molecular structure. A 15-carbon (C6–C3–C6) backbone with two phenyl units (A and B), along with a heterocyclic unit (C), is the characteristic of flavonoids. Non-flavonoid polyphenolic compounds include phenolic acids, coumarins, lignans, hydrolyzable tannins, lignins, and condensed tannins. Honey contains a wide range of both flavonoids and phenolic acids [10].

Among flavonoids, quercetin, myricetin, kaempferol, luteolin, rutin, naringenin, naringin, chrysin, rhamnetin, isorhamnetin, apigenin, pinocembrin, pinobanksin, galangin, tricetin, catechin, and hesperidin are the major compounds found in various varieties of honey. The most common phenolic acids, occurring in almost every honey type, include caffeic acid, gallic acid, coumaric acid, syringic acid, cinnamic acid, ferulic acid, chlorogenic acid, ellagic acid, benzoic acid, vanillic acid, phenylacetic acid, and homogentisic acid. In honey, though polyphenols are among the minor components, they are the main explanation for various health benefits honey. Several in vitro and in vivo studies have demonstrated the beneficial effects of these flavonoids and non-flavonoids in various diseases and maladies [11,12,13,14]. Since the taste, color, and composition of various kinds of honey depend upon the type of floral origin, the geographical area, and the various species of bees engaged in honey production, the qualitative and quantitative differences in the polyphenolic contents of various types of honey have been determined in various studies that may explain the diverse pharmaceutical properties of these different types of honey (Table 1).

## 3. Medicinal Properties of Honey

In addition to its nutritive value, honey possesses a wide variety of therapeutic properties. It has been used to treat several diseases since ancient times [25,26]. Its most common application is for healing wounds and skin infections [27,28,29]. Honey possesses significant antibacterial [30,31,32], antiviral [33,34], antifungal [27,35,36,37], antioxidant [27,38,39,40], anti-inflammatory [41,42,43], antineoplastic [28,44], antimicrobial [45,46,47], anticarcinogen [48,49,50], antiarrhythmic [51,52], antileishmanial [53,54], antithrombotic, antiplatelet [55,56], antimutagenic [57,58], antinociceptive [59,60], antimycobacterial [7,61], antiproliferative [62,63], and immune-boosting [64,65,66] properties. It is also shown to have hypocholesterolemic [67,68], cardioprotective [69,70], antihypertensive [71], hepatoprotective [72,73], gastroprotective [41,74], neuroprotective [75,76], nephroprotective [77,78], and hypoglycemic [79,80] effects. The literature suggests that honey is quite useful to overcome complications of the reproductive system in both males and females. It is shown to improve spermatogenesis [81] and improve sperm count and their motility [82]. It is shown to protects against vaginal and uterine atrophy [83] and improves the normal estrus cycle [84].

## 4. Honey and Neurological Disorders

Neurological disorders are ailments of the central and peripheral nervous systems. Neurological disorders have become one of the major health issues, particularly in the recent modern era. The burden of neurological disorders has increased dramatically in the last few decades throughout the world, from third-world countries to the world’s most developed countries [85]. Among neurological disorders, neurodegenerative diseases such as Alzheimer’s disease (AD), Parkinson’s disease (PD), Huntington’s disease (HD), multiple sclerosis (MS), and stroke are the most commonly prevalent maladies. By and large, the *dysfunction* or death of nerve cells in specific areas of the brain is the main cause of all these diseases.

The leading underlying cause of nerve death is oxidative stress caused by an accumulation of free radicals and depletion of antioxidants [86]. Increasing the levels of antioxidants can be beneficial against these neurodegenerative diseases [24]. Honey contains antioxidants in the form of polyphenols and flavonoids. All kinds of honey contain these bioactive compounds but in varying concentrations. Various colorimetric assays reveal that the total phenolic content in various types of honey varies from 86 mg/kg to 1141 mg/kg [87], whereas the range for the flavonoid content is from 36 mg/kg to 150 mg/kg of honey [88]. Both flavonoids and polyphenols protect neurons against oxidative damage, improve neuronal function and enhance regeneration, protect neurons from neurotoxicity, and modulate neuronal signaling pathways [24].

**Figure 1:**
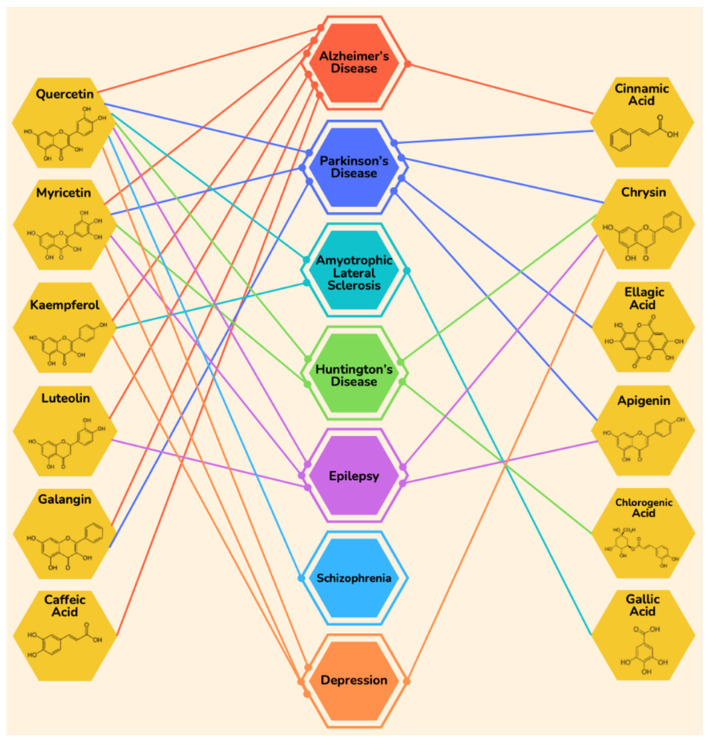
The therapeutic potential of various polyphenols in honey in different neurological disorders. Various types of honey have a wide range of these vital polyphenols, suggesting honey as a potent complementary and alternative medicine for the management and treatment of a variety of neurological diseases.

The most common flavonoids and polyphenols present in almost all types of honey that are found to be beneficial against neurodegenerative diseases include apigenin, benzoic acid, caffeic acid, catechin, chlorogenic acid, chrysin, cinnamic acid, coumaric acid, ellagic acid, ferulic acids, galangin, gallic acid, hesperetin, isorhamnetin, kaempferol, luteolin, myricetin, naringenin, quercetin, and syringic acid [88] (Figure 1). 

Though plenty of research is available in the literature archive advocating the potential role of honey in ameliorating neurological disorders, the polyphenols components of honey proclaim way more potential roles of sweet nectar. In this review, we have indexed and comprehended the role of various polyphenols that are found in honey with their reported and potential role in the prevention, management, and treatment of various neurological disorders (Table 2). 

### 4.1. Alzheimer’s Disease

Alzheimer’s disease (AD) is a very common age-related neurodegenerative disease. The pathology of the disease includes the formation of neuritic plaques, mainly composed of the β-amyloid (Aβ) peptide, along with neurofibrillary tangles [185,186]. Studies suggest oxidative stress as the underlying cause of these neuronal malfunctions in AD [187,188]. Membranes of brain cells are highly sensitive to oxidative damage because of high levels of polyunsaturated fatty acids [189,190]. The excessive production of reactive oxygen species causes mitochondrial damage and decreased ATP production in AD. Many preclinical investigations suggest that oxidative stress causes neuroinflammation and stimulates Aβ production. Neuronal cell dysfunction and death are the ultimate results of these processes [97,191]. In the literature, honey has been reported to protect the astrocytes from this oxidative damage. Astrocytes serve as the major site for the expression of genes causative of Alzheimer’s disease [192,193]. 

Different varieties of honey have been reported to act as a potential natural source of acetylcholinesterase (AChE) inhibitors in Alzheimer’s disease. Acetylcholinesterase activity is associated with a depletion of acetylcholine which results in memory-related problems in Alzheimer’s disease. Therefore, the inhibition of acetylcholinesterase (AChE) is a possible therapeutic strategy to deal with this disorder [194,195]. The co-administration of honey syrup and an aqueous saffron extract is shown to increase the level of antioxidant enzymes, such as superoxide dismutase (SOD), catalase (CAT), and glutathione peroxidase (GSH-Px), and mitigate oxidative stress in aluminum chloride-induced neurotoxicity. Aluminum is an environmental factor that is reported to alter several oxidative enzymes and other biomolecules related to neurotoxicity and Alzheimer’s disease (AD) [196]. Another hallmark of Alzheimer’s disease is the loss of cortical and hippocampal neurons due to reduced cerebral blood flow, which consequently leads to cognitive impairments. A study by Saxena et al. revealed that the daily supplementation of Tualang honey to chronic cerebral hypoperfusion induced by permanent bilateral common carotid arteries ligation (2VO) in rats efficiently reduced neuronal cell loss and increased the viable neurons [197]. In addition to honey as a whole, several studies have been registered in the literature archive regarding the potential beneficial effects of specific components of honey in AD patients.

Quercetin is well known to improve the mitochondrial activity in living cells [89]. Pre-treatment with quercetin decreases oxidative stress by the activation of the Nrf2 signaling pathway in β-Amyloid-induced models of Alzheimer’s disease [90]. Nrf2 signaling is involved in balancing the levels of antioxidant enzymes, and its activation serves as the marker for the amelioration of neurodegeneration in AD models [198,199,200,201]. Furthermore, quercetin also shows a neuroprotective effect in AD models via the regulation of PON2 activity [91]. The PON2 protein, a member of the paraoxonase family, is involved in reducing oxidative stress. Being localized mainly in mitochondria, PON2 decreases oxidative stress by preventing the formation of superoxide, a free radical, at the inner mitochondrial membrane [202]. Quercetin has also been reported to cause a reduction in neuroinflammation and neurodegeneration, a decrease in astrogliosis, and recovery in cognition disabilities in mouse models of AD [92,93].

Myricetin, a flavonoid present in honey, has been shown to have a protective effect on neurons present in the hippocampus, a part of the brain involved in memory processes. Myricetin reverses the impairments of cognitive functions and improves the memory in streptozotocin-induced models of AD [94]. It is reported to exhibit an anti-tau protein effect and preventive effect against the formation of amyloid-beta aggregates in AD [95]. In addition to that, myricetin reduces the activity of acetylcholinesterase as well as increases the concentration of acetylcholine in the hippocampus. Treatment with myricetin ameliorates the damage caused by oxidative stress in the hippocampal region of mice. It does so by increasing the levels of antioxidative enzymes [96]. Myricetin also indicates anti-inflammatory activity via the inhibition of the NF-κB pathway and NLRP3 inflammasome [97,98]. The formation of amyloid-beta fibrils is an important hallmark of AD. Myricetin inhibits not only the formation of fibrils of amyloid-beta, but also the oligomers of amyloid-beta [99].

Kaempferol, another flavonoid present in honey, has also been reported to improve the cognitive deficits in the drosophila model of AD. Beg and colleagues reported that kaempferol can inhibit the formation of senile plaques which are characteristics of AD. It decreases the level of lipid peroxidation in drosophila which increased due to AD. Kaempferol also protects the cell from oxidative and apoptotic damage [100]. Furthermore, it has also been demonstrated that kaempferol mitigates cognitive dysfunctions by controlling oxidative damage and neuroinflammation in a model of sporadic dementia. It regulates the concentrations of antioxidative enzymes, such as superoxide dismutase (SOD), and causes a reduction in the levels of tumor necrosis factor-α (TNF-α), a pro-inflammatory cytokine [101]. Kaempferol has also been reported to impart a neuroprotective effect against AD via anti-apoptotic activity as well. It causes the downregulation of genes associated with apoptosis, such as Bcl-2-associated X protein (Bax) and cleaved caspase-9 in SH-SY5Y neuronal cells treated with amyloid-beta (Aβ) [102]. It has been observed that the density of neurons decreases in the CA1 region of the hippocampus of streptozotocin-induced rat models of AD. However, in treatment with kaempferol, an elevation in the density of neurons is seen in the same region [103]. Kaempferol has also been reported to downregulate the expression of proteins that are associated with inflammation, such as interleukin-1β (IL-1 β), inducible nitric oxide synthase (iNOS), and cyclooxygenase 2 (COX-2), in amyloid beta-induced models [104]. Kaempferol reduces the level of intracellular oxidative stress and protects the cell from damage [105].

Luteolin, another antioxidant, possesses a strong potential against Alzheimer’s disease. This potential is attributed to its capacity to inhibit the formation of amyloid-beta and tau protein aggregates. It causes the downregulation of various inflammation- and apoptosis-associated proteins such as tumor necrosis factor α (TNF-α), cyclooxygenase 2 (COX-2), and interleukins. It ameliorates oxidative stress by scavenging the free radicals. Luteolin also modifies the functions of various transcription factors such as NF-Kb, p53, cJun, p53, Nrf-1, β-catenin, and AP-1 [106]. Luteolin improves deficits of memory and averts the decrease in density of the cell layer in the CA1 hippocampal region of streptozotocin-induced AD models [107]. Galangin, a flavanol component of honey, diminishes the cytotoxicity induced by okadaic acid in PC12 cells. It decreases the levels of p-tau, β-secretase, and Aβ42. Galangin diminishes autophagy by increasing the phosphorylation of Akt and mTOR and ameliorating the phosphorylation of GSK3β. Both factors contribute to the inhibition of autophagy in okadaic acid (OA)-induced PC12 cells [108]. Galangin has also been demonstrated to improve cognitive and memory functions as well as increase the concentration of acetylcholine in scopolamine-induced memory impairment models [109].

Caffeic acid, a phenolic acid present in honey in significant amounts, reduces inflammation, the activity of acetylcholinesterase, and oxidative stress in models of AD. It regulates the expression of p53, MAPK, p38, and caspase 3. These anti-oxidative and anti-inflammatory properties of caffeic acid make it suitable for use against AD [110]. Cinnamic acid, another bioflavonoid present in honey, is shown to attenuate the formation of amyloid plaques and to improve the memory impairments in 5XFAD mice through lysosomal biogenesis via the activation of peroxisome proliferator activating receptor α (PPARα), a member of the nuclear receptors’ family [111].

### 4.2. Parkinson’s Disease

Parkinson’s disease (PD) is the second most widespread and chronic neurological disorder in the present world. It is a neurodegenerative disease, mainly characterized by the degeneration of dopaminergic neurons in substantia nigra pars compacta (SNc), a part of the midbrain. This degeneration happens mainly because of the formation of Lewy bodies. Lewy bodies are cytoplasmic inclusions of misfolded proteins, primarily the α-synuclein, along with ubiquitin and others. Rigidity, tremor, bradykinesia, postural instability, and gait problems are the basic clinical features attributed to PD. Non-motor symptoms associated with PD include sleep difficulty, depression, and deficits of olfaction. The death of neuronal cells in PD is mediated by many factors which include oxidative stress, endoplasmic stress, dysfunction of mitochondria, defects in the process of autophagy, and inflammation. Oxidative stress results from the accumulation of reactive oxygen species (ROS) and a lack of antioxidants [203,204,205,206,207,208,209,210]. Since this oxidative stress is the key player behind neurodegeneration, the use of antioxidants is a common therapeutic strategy against PD [211]. 

Honey contains a variety of antioxidants in the form of flavonoids and phenolic acids, which can potentially reduce oxidative stress and repair the damage caused by it. Honey, along with other apitherapeutic products, has been reported to inhibit the activity of monoamine oxidase (MAO), which is involved in reducing the free radical scavenging activity and causing oxidative damage in neurodegenerative disorders such as Parkinson’s disease [212]. Recently a study by Topal et al. reported that Parkinson’s patients can make use of only pollen and honey produced by those bees that feed on flowers of Vicia faba L. as a treatment for Parkinson’s disease. This kind of honey contains a considerable percentage of L-DOPA, a commonly employed drug against Parkinson’s [213]. In conjunction with these studies, the polyphenols present in honey are also well studied individually for their use against neurodegenerative diseases.

Quercetin, a flavonoid present in almost all kinds of honey, has been reported to protect neuronal degeneration in substantia nigra and increase dopamine levels in MitoPark mice. It also initiates the activation of various kinases such as PDK1 and Akt which are important for the survival of neuronal cells. Further, it enhances the expression of BDNF, ameliorates mitochondrial dysfunction, and upsurges the energy production by the mitochondria [112]. Quercetin causes the restoration of activities of antioxidant enzymes and repairs the cognition deficits related to PD in the 6-hydroxydopamine-induced model of Parkinson’s disease [113]. El-horany et al. demonstrated that quercetin shows a neuroprotective effect in the rotenone-induced rat model of Parkinson’s disease via decreasing the endoplasmic reticulum stress and boosting the process of autophagy. It does so by decreasing the C/EBP homologous protein (CHOP) which is related to ER stress and increasing the expression of Beclin-1, which activates autophagy [114]. Myricetin, another flavonoid, is also considered very beneficial against Parkinson’s disease. It is reported to not only increase the levels of dopamine in a dose-dependent manner, but also prevent the degeneration of dopaminergic neurons in the transgenic drosophila model of Parkinson’s disease [115]. A study on LPS-induced models of PD reveals that myricetin employs considerable neuroprotection in dopaminergic neurons via the inhibition of inflammation. It ameliorates the changes in motor behaviors caused by PD and prevents the loss of neurons by preventing the activation of microglia. It also diminishes the activation of inflammation-associated cytokines (IL-1 β, TNFα, and IL-6) and some signaling pathways such as MAPK and NF-Κb [116].

Chrysin, a flavonoid abundantly present in honey, is well studied for its role in PD. Chrysin has been reported to exhibit a neuroprotective effect against the MPTP-induced loss of dopaminergic neurons in mice by increasing the expression of the survival factor MEF2D via the AKT/GSK3β/MEF2D pathway. It also causes the inhibition of MAO-B [117]. Chrysin is found to ameliorate the oxidative damage, neuroinflammation, and dysfunction of Na+, K+-ATPase activity in the 6-hydroxydopamine mice model of Parkinson’s disease [118]. Chrysin improves the condition of neuron loss and alterations in motor functions along with improved memory processes in rotenone-induced rat models of PD [119]. 

Ellagic acid, another polyphenol, is shown to exert an inhibitory effect on monoamine oxidase (MAO) activity which results in decreased oxidative stress and prevents the loss of dopaminergic neurons [120]. Sarkaki et al. advocated the use of ellagic acid against PD as it protects neurons from oxidative damage induced by 6-OHDA via modulating the levels of antioxidants [121]. A study on ellagic acid revealed that ellagic acid causes the extenuation of apoptosis and reduces oxidative stress by enhancing the antioxidant defense system in the 6-OHDA-induced model of Parkinson’s disease. Additionally, it also causes the inhibition of MAO-B and modulates the ERβ/Nrf2/HO-1 signaling cascade. Altogether, these findings support the potential neuroprotective role of ellagic acid in PD patients [122].

Cinnamic acid, another bioflavonoid present in honey, is known to be effective against PD in the MPTP-induced model. It protects neurodegeneration in substantia nigra via the activation of peroxisome proliferator activating receptor α (PPARα), which exerts a neuroprotective role in multiple ways [123]. Galangin, the flavonol class member, is reported to prevent the loss of dopaminergic neurons in LPS-intoxicated mice. It inhibits microglial activation via AKT, NF-κB p65, JNK, and p-38 signaling association and suppresses the formation of inflammation-related factors [124]. Choi et al. demonstrated that galangin is an anti-inflammatory agent in microglia stimulated by LPS. It causes the activation of PPAR-γ, which is shown to be neuroprotective in PD through mitigating inflammation, mitochondrial dysfunction, the inhibition of apoptosis, and oxidative stress [125,126,127]. Apigenin, another component of honey, has been described to be a neuroprotective agent against PD in the transgenic Drosophila model. Its neuroprotective role is attributed either to its antioxidative property through the inhibition of MAO, or anti-apoptotic activity via the inhibition of caspase-3 activation [128].

### 4.3. Huntington’s Disease

Huntington’s disease (HD) is another genetic, progressive neurodegenerative disease prevalent all around the globe. The main cause of the disease is the repeated expansion of the CAG trinucleotide in the huntingtin gene (HTT) present on chromosome 4. It leads to the production of the mutant huntingtin protein (mHTT) which contains an abnormally long tract of polyglutamine [214,215,216]. The penetrance of the disease increases with age and is transferred to the next generation in an autosomal dominant manner. Symptoms of this disease include a decline in motor functions, cognitive dysfunctions, and psychological disturbances, which include both neural dysfunction and, ultimately, cell death. These neurodegenerative changes initially began in the striatum and gradually spread to other areas such as the hippocampus and cortex [130,217]. Oxidative stress is considered the key player in the occurrence and progression of the disease [130,218]. Due to the involvement of oxidative stress, the use of antioxidants is believed to be highly beneficial against HD. Like other neurodegenerative diseases, antioxidants present in honey have been extensively investigated in several studies for their potential benefits in HD. 

Quercetin is shown to be involved in the protection of neurons in a 3-NP-induced model of HD. It causes a decrease in oxidative stress by modulating antioxidant systems. It also elevates the energy production of mitochondria via the regulation of peroxisome proliferator-activated receptor-gamma coactivator (PGC-1) or sirtuins (SIRT1). Additionally, it has also been found helpful in the improvement of motor functions of neurons [129]. Quercetin is reported to ameliorate the neurochemical, neuropathological, and behavioral malfunctions induced by 3-nitropropionic in rats. It causes the suppression of monoamine oxidase A (MAO-A) activity, which exerts an overall anxiolytic effect [130]. Another important flavonoid, myricetin, along the same lines, has been described to be neuroprotective against HD. Myricetin reduces the aggregation of polyglutamine in HD patients [131]. It decreases the proteo-toxicity caused by polyglutamine aggregates and repairs the behavioral impairments in 3-NP-induced models of HD [132].

Chrysin shows antioxidant and anti-apoptotic activity in the 3-NP-induced model of HD. It causes an increase in the levels of antioxidant enzymes. It also causes the downregulation of pro-apoptotic factors such as Bax, along with the upregulation of anti-apoptotic factor Bcl-2 [133]. A recent study by Haider et al. demonstrates that chrysin shows a neuroprotective effect against the 3-NP-induced model of HD. It causes the restoration of neurobehavioral functions and modulates motor functions through increasing serotonin levels and monoamine oxidase (MAO) activity. Furthermore, it has also been reported to protect the degeneration of striatal neurons [134]. On the other hand, another important component of honey, chlorogenic acid, has also been reported to protect the cells from toxicity and genotoxicity induced by 3-NP [135].

### 4.4. Amyotrophic Lateral Sclerosis

Amyotrophic lateral sclerosis (ALS) is another neurodegenerative disease that adversely affects the nerve cells in the brain and spinal cord, resulting in a loss of muscle control. It is an adult-onset, fatal, paralytic, and progressive degenerative disease, primarily of motor neurons. It involves the formation of intracellular aggregates of the TAR DNA-binding protein 43 (TDP-43) protein. Expansions of a hexanucleotide repeat in C9orf72 serve as the main genetic source of ALS. It can be either inherited, known as familial amyotrophic lateral sclerosis, or sporadic. The formation of aggregates of enzyme Cu-Zn superoxide dismutase (SOD) is a common feature of both forms. It begins initially with focal weakness, but spreads persistently to most muscles, together with the diaphragm. Almost half of the patients with ALS switch to develop behavioral and cognitive dysfunction [138,219,220,221]. Neurodegeneration in ALS is mainly attributed to oxidative stress and, therefore, antioxidant therapy may help fight against the disease [222].

Another major hallmark in the early onset of amyotrophic lateral sclerosis is the mutations in the gene encoding ubiquitin chaperone *Ubiquilin 2* (*UBQLN2*), which causes defects in ubiquitin-binding abilities and results in the restricted delivery of ubiquitinated substrates to proteasomes for degradation [223]. A study by Phokasem et al. revealed that coffee honey restored locomotor capacity, increased learning ability, and mitigated oxidative stress in the brain of the *Ubiquilin*-knockdown *Drosophila* model [224].

Quercetin, an important flavonoid component of honey, is evaluated for its neuroprotective effect against ALS. Derivatives of quercetin were shown to have an inhibitory effect on the aggregation of the Cu-Zn superoxide dismutase (SOD) enzyme [136]. Recently, quercetin itself has been studied for its role in ALS. It is shown to inhibit the aggregation of the Cu-Zn superoxide dismutase (SOD) enzyme. It binds to monomeric and oligomeric forms of the SOD enzyme, thus preventing self-association as well as the elongation of fibrils of the SOD enzyme [137]. Kaempferol, another flavonoid, shows protection against neurotoxicity induced by mutant SOD1 in a model of ALS. Kaempferol prevents cell death and reduces the aggregation of intracellular SOD1. It boosts up the process of autophagy, a way of eliminating misfolded protein aggregates, via the AMP-activated protein kinase (AMPK)-mTOR pathway. It increases AMPK phosphorylation which inhibits mTOR phosphorylation, and subsequently induces autophagy [138,139].

Coumaric acid is another important component of honey that is shown to be beneficial against ALS. It is shown that p-coumaric acid exerts a neuroprotective effect against mutant SOD1-induced neurotoxicity. It ameliorates oxidative stress and endoplasmic reticulum stress. It also enhances the process of autophagy [140]. Gallic acid has also been suggested to be effective against sporadic amyotrophic lateral sclerosis. It boosts up the activities of antioxidant enzymes and, at the same time, decreases lipid peroxidation levels. It declines the process of inflammation via the downregulation of inflammation-related factors such as TNF-α, IL-6, IL-β, and NF-κB in the quinolinic acid-induced neurotoxicity model [141]. Gallic acid is shown to improve motor functions and motor learning abilities in a dose-dependent manner in the aluminum-induced neurodegeneration model. It prevents glutamate excitotoxicity and maintains antioxidant status. It inhibits the formation of neurofibrillary tangles, thus protecting neurons from damage [142]. Another study by Aaron and colleagues has also suggested gallic acid to be beneficial against ALS. It is shown to improve motor skills and decrease TDP-43 proteotoxicity in a C. elegans model of ALS [143].

### 4.5. Epilepsy

Epilepsy is a serious neurological disorder that is known to affect about 70 million people globally. It exhibits a bunch of symptoms rather than a single complaint. It is categorized by a long-term predisposition that results in spontaneous epileptic seizures and leads to several neurobiological, psychosocial, and cognitive consequences. Epileptic seizures are persistent convulsive events characterized by conventional behavioral variations that show the basic neural mechanisms related to the disease [225,226]. Oxidative stress, inflammatory factors, and the unnecessary production and release of excitatory amino acids are known to play a key role in the pathology of epilepsy [227]. Many antiepileptic drugs are available nowadays, but these are ineffective in controlling epileptic seizures in about 30% of individuals suffering from epilepsy. Therefore, numerous approaches have been developed to produce novel medications for the treatment of epilepsy and a great emphasis is put on the use of natural products against epilepsy to reduce the side effects of chemical drugs [228,229]. 

The abundance of antioxidants present in honey has proven to exhibit beneficial roles against epilepsy. Quercetin is shown to have an ameliorating effect on epilepsy. It is shown to exert anti-inflammatory activity in kainic acid (KA)-induced models of epilepsy. It inhibits the activation of microglial cells and the subsequent release of inflammatory cytokines such as NF-κB, TNF-α, and IL-1β. It has also been reported to attenuate the seizures induced by KA. All these factors endorse an overall protective effect of honey against epilepsy [144]. Quercetin is reported to inhibit the expression of a gene for β subunits of GABA receptors which attenuates the progression of neurodegeneration in the KA-induced model of the seizure [145]. Quercetin supplementation, along with levetiracetam, reduces the depression associated with epilepsy. Depression in epilepsy is mainly caused by the predisposition of tryptophan levels. Quercetin restores tryptophan levels, thus diminishing the epilepsy comorbid depression [146]. Quercetin exerts a strong anticonvulsant effect in the maximal electric shock (MES) induced model of seizures in a dose-dependent manner. It does so by modulating the activities of glycinergic and GABAergic ion channels [147]. Quercetin-loaded nanoparticles exhibit anticonvulsant activity via reducing the behavioral signs of seizures, loss of neurons, and astrocyte activation in a pentylenetetrazol (PTZ)-induced kindling model [148].

Myricetin ameliorates the intensity of seizures and protects neuronal loss in the pentylenetetrazol-kindled mice model of epilepsy. It reduces the degeneration of neurons and inhibits apoptosis via the downregulation of Bad, Bax, and cleaved caspase-3 expression levels, along with the upregulation of anti-apoptotic proteins such as Bcl-2 and Bcl-xL. Myricetin normalizes the glutamate/GABA ratio which gets disturbed during epilepsy [149,150]. Luteolin has also been reported to be effective against epilepsy. Luteolin pre-treatment revokes the seizure elated cognitive deficits and reduces the severity of seizures in pentylenetetrazol-kindled mice. It causes an enhanced expression of Brain-Derived Neurotrophic Factor (BDNF), a factor essential for neuronal survival. It has also been reported to help in the mitigation of oxidative stress. Both these factors contribute to the improvement of cognitive functions [151]. Tambe et al. demonstrated that luteolin exerts an anticonvulsant effect by increasing the activation of receptors for GABAA, which facilitates the opening of chloride ion channels, and by enhancing the seizure threshold. Luteolin not only inhibits the production of seizures, but also diminishes oxidative stress via its free radical scavenging activity. These aspects support the anti-epileptogenic activity of luteolin and make it a potential candidate for epilepsy [152]. Luteolin could efficiently improve the cognition impairments in epileptic rats, and the underlying mechanism might be related to modulating the CaM-CaMPK signaling pathway via the downregulation of CaM, CaMPK, as well as the Ras protein [153].

Chrysin, another flavonoid present in honey, also exerts a neuroprotective effect against epilepsy. Chrysin attenuates the seizures induced by pentylenetetrazol in experimental models. It exhibits the anticonvulsant activity via the modulation of GABAA receptors and abrogates the convulsion-induced oxidative damage [154]. Chrysin-loaded nanoparticles lessen the severity of epileptic seizures and ameliorate the oxidative damage in the brains of pentylenetetrazol-kindled rats. Further, these particles reduce the process of apoptosis by boosting the expression of Nrf2, NQO-1, and HO-1. It supports that chrysin can act as a possible therapeutic approach to improve neurodegeneration in epilepsy [155]. Apigenin, another flavonoid, has also been suggested to be an effective neuroprotective agent against epilepsy. Apigenin employs a protective effect on kainite-induced memory impairments via an anticonvulsant and anti-apoptotic function. It reduces the neuronal loss in the hippocampus and the release of cytochrome c from the mitochondria which leads to the alleviation of apoptosis in the mitochondria [156]. Apigenin abrogates the myeloperoxidase-induced oxidative damage in the kainic acid-induced model of epilepsy. It inhibits the overexpression of hypochlorite (HClO). The production of hypochlorite (HClO) by myeloperoxidase is contemplated to be closely related to the development of epilepsy [157,158]. Sharma et al. revealed that apigenin averts the cognitive impairments, hence repairing the behavioral deficiencies, and exerts antidepressant and anti-anxiolytic effects in pentylenetetrazol-induced kindling models of epilepsy. These effects can be ascribed to an increased expression of BDNF via enhanced CREB and serotonin levels in the hippocampal region. Both serotonin and CREB are involved in the expression of the BDNF gene [159,230,231].

Ferulic acid, a phenolic acid, is another important component of honey that is effective against epilepsy. Supplementation of ferulic acid along with levetiracetam, an epileptic drug, palliates the epilepsy-associated depression in pentylenetetrazol-kindled animal models of epilepsy. Increased levels of proinflammatory cytokines and enhanced cyclooxygenase 2 (COX2) activity led to the upregulation of indoleamine 2,3-dioxygenase (IDO) activity and, consequently, causes depression in epileptic individuals [232,233]. Ferulic acid exerts an antidepressant effect via decreasing the levels of proinflammatory cytokines such as TNF-α and IL-1β, the inhibition of cyclooxygenase 2 (COX2) activity, the modulation of the corticosterone level, and repairing hypothalamus pituitary adrenal (HPA) axis dysfunction [160]. In another study, ferulic acid showed neuroprotective effects in models of epilepsy using pentylenetetrazol through the amelioration of oxidative stress and the upregulation of neuroprotective heat shock protein 70 (Hsp70), along with neurotransmitters such as serotonin (5-HT) and norepinephrine (NE) [161]. Hassanzadeh et al. reported that ferulic acid exerts an anti-epileptogenic effect via diminishing the oxidative stress, repairing the cognitive deficits, and decreasing the seizure activity [162]. Ferulic acid has also been shown to improve memory functions and learning capacities as well. Pre-treatment of animals with ferulic acid prevents the entylenetetrazol-induced oxidative, as well as cognitive, damage. It inhibits the apoptotic process via the activation of anti-apoptotic protein Bcl-2 and scavenges the free radicles to ameliorate oxidative damage [163].

Naringenin, another flavonoid, is a potential therapeutic agent against epilepsy. Naringenin exhibits a neuroprotective role against pilocarpine-induced epilepsy models. Pilocarpine causes the generation of seizures via the production of free radicals, which affects the respiratory chain inside the mitochondria, weakens the lysosomal membranes, and decreases the threshold for convulsions. Naringenin works as a strong antioxidant against pilocarpine-induced seizures. It causes an elevation in the levels of antioxidant enzymes, restores neuronal morphology, and reduces the neurodegeneration in the hippocampal area [164,234]. Granule cell disruption (GCD) occurring in the dentate gyrus (DG), an area of the hippocampus, is a pathological feature of temporal lobe epilepsy [235]. Naringenin is shown to impede the occurrence of seizures and decrease the kainic acid-instigated GCD in the hippocampus. Further, it ameliorates the generation of proinflammatory cytokines such as tumor necrosis factor-α (TNFα) and interleukin-1β (IL-1β), which plays an antiepileptic role [165]. Naringenin shows an anticonvulsant effect in pentylenetetrazol- and MES-induced epilepsy models. This anticonvulsant role of naringenin is attributed to its antioxidant effect, agonist effect on GABAA receptors, and reduction of glutamate transmission [166].

### 4.6. Schizophrenia

Schizophrenia is a chronic, devastating neurodegenerative disorder known to affect more than 1% of the population across the globe. It causes the impairment of mental and social functions and often results in various comorbid diseases. Both men and women are affected in equal numbers. It is labeled by negative and positive symptoms. Hallucinations and delusions are the main positive symptoms of this disease, whereas negative symptoms include social withdrawal or isolation, lack of emotional expressiveness, and depression [168,236]. Instabilities of main cognitive processes such as executive functions, attention, and certain forms of memory (particularly working memory) are common among patients of schizophrenia, that combinedly lead to the behavioral alterations and functional disturbances of schizophrenia. Both genetic and environmental factors are involved in the development of this disorder [237].

Oxidative stress is an important underlying mechanism in the progression of the disease because the central nervous system is more susceptible to oxidative stress in comparison to other body organs. To combat oxidative damage, the use of antioxidants is a common and useful therapy against schizophrenia [238,239]. A study by Yahaya et al. showed that supplementing tualang honey to schizophrenic individuals for 8 weeks greatly affected their working memory. Tualang honey acts as a cognitive enhancer and improves learning abilities and cholinergic abnormalities in schizophrenia [240]. Antioxidants present in honey are also known to play vital roles against schizophrenia. Quercetin improved the cognitive impairments in a ketamine-induced mice model of schizophrenia. It exerts its effect via scavenging free radicals and increasing the levels of antioxidant enzymes [167]. Quercetin has also been reported to reduce the depressive behaviors in ketamine-instigated models of schizophrenia [168]. It also improves the behavioral impairments in schizophrenic models [169]. A case study report by Schwartz suggests that quercetin can be effectively used to boost antipsychotic therapy [170].

### 4.7. Depression

Depression is a serious mental illness that is known to affect more than 465 million people worldwide. According to the World Health Organization, depression is the second leading cause of disabilities across the globe. According to the American Psychological Association, depression is characterized by a lack of interest in nearly all activities, alterations in appetite, disturbed sleep, disordered psychomotor functions, feeling of uselessness and guilt, decreased decision-making abilities, thoughts of suicidal plans, and even suicidal attempts [241]. It is a complex pathophysiological condition that involves a diminution of the monoamine system, specifically in the neurotransmission of neurotransmitters including dopamine, serotonin (5HT), and norepinephrine, or the diminished functions of receptors for these neurotransmitters [242]. Impairments in functions of these neurotransmitters, mainly serotonin (5HT) and norepinephrine, lead to the development of depression [243,244,245]. Dysfunction of the immune system, both innate and adaptive, also occurs in depression. Elevation in the levels of inflammatory proteins and lower levels of BDNF are observed in patients with a mood disorder [246,247,248]. Oxidative stress is also considered a key player in the pathogenesis of the depressive disorder. Therefore, the use of antioxidants is considered an effective strategy against depression [249,250,251,252,253].

Honey is shown to be an effective prescription against depression owing to its antioxidant properties [254]. Polyphenols present in honey are considered an effective therapy in the cure and management of depressive disorders. Quercetin has been studied to exert antidepressant effects in the unpredictable chronic mild stress (UCMS) model of depression. It causes an increase in the levels of antioxidant enzymes, a decrease in the levels of inflammatory factors, and accrues serotonin (5HT) levels [171,172]. In a model of LPS-induced depression, quercetin mitigates depressive behavior by modulating levels of BDNF and its related factors [173]. Myricetin is also found useful in combating mood disorders. It recovers hopeless behavior in mice exposed to restraint stress for 21 days [174]. These protective effects are mainly due to the regulation of BDNF levels in the hippocampus and its antioxidant ability [175]. Kaempferol is reported to exert anti-depressive effects in a chronic social defeat stress (CSDS) mouse model. It causes an increase in the levels of antioxidant enzymes, decreases the levels of inflammatory cytokines, and upregulates the AKT/β-catenin cascade to overcome depression-like behaviors [176].

Chrysin, another flavonoid, is of major importance against depression. In mice subjected to chronic unpredictable mild stress (CUMS), chrysin is shown to exert protective effects through the upregulation of the nerve growth factor (NGF) and BDNF. It also normalizes Na+, K+-ATPase activity, which is compromised during the depression [177]. It employs anti-depressant effects in a model of agitated depression via the inhibition of the kynurenine pathway, the downregulation of inflammatory cytokines, and elevation in the levels of serotonin (5HT) and BDNF [178]. Naringenin exhibits potent anti-depressant effects. Bansal et al. reported that naringenin is quite beneficial against depression-like behaviors in the olfactory bulbectomized mice model of depression. It performs anti-depressive activity via the restoration of antioxidant enzymes’ levels, the elevation of BDNF levels, the modulation of serotonin levels, and the downregulation of inflammation mediators [179]. It alleviates depression-like behavioral instabilities caused by social defeat stress in mice through the inhibition of acetylcholinesterase activity, oxidative damage, and the release of inflammatory proteins [180]. It also causes the upregulation of BDNF, NKX2, PAX6, and sonic hedgehog (Shh) signaling to overcome the depression induced by CUMS [181].

Coumaric acid, a phenolic acid, has also been suggested as a potential therapeutic agent against depression. It improves behavioral hopelessness, lessens inflammation-associated alterations, enhances neurotrophic activity, and mitigates long-term synaptic depression (LTD) in the hippocampus induced by LPS [182]. Ferulic acid, another phenolic acid, also exhibits anti-depressive properties. In offspring rats subjected to prenatal stress (PS), ferulic acid treatment works as an anti-depressant. PS causes the upregulation of pro-inflammatory cytokines, including TNF-α, IL-1β, IL-6, and the expression of NF-κB and iNOS in the hippocampus which leads to the development of depression. Ferulic acid reverses all these changes and abrogates depression-like behaviors [183]. It has also been reported to ameliorate the depressive behaviors induced by LPS by downregulating the factors associated with inflammation and apoptosis [184].

## 5. Conclusions

Honey is a miraculous natural product with wonderful nutritional and therapeutic potential. It has been an important part of ethnomedicine since ancient times in every human civilization and culture for the cure and treatment of several maladies. In modern history, liquid gold has become an appreciable component of contemporary medicine used along with standard medical treatment for various ailments. Polyphenols present in honey have been proven to play vital roles against different neurological disorders, including Alzheimer’s disease, Parkinson’s disease, Huntington’s disease, Depression, etc. Nevertheless, further studies are needed to investigate the effects of honey, either individually or in combination with other complementary and alternative medicines, against various prevalent neurological disorders.

## 6. Limitations and Future Perspectives 

Recent approaches toward the discovery of novel therapies for neurodegenerative disorders can be classified into four categories, namely protection against oxidative damage, reduction in neuroinflammation, the inhibition of enzymes that degrade neurotransmitters, and protection against neurotoxic environmental factors. All the studies included in the data cover the abovementioned approaches and provide comprehensive evidence in terms of the neuroprotective potential of polyphenols, but these are not enough to make a judgment about their clinical benefits. Moreover, there is a dearth of studies related to the direct use of honey against neurodegenerative diseases. Only a few studies on the direct use of honey against Alzheimer’s disease, Parkinson’s disease, amyotrophic lateral sclerosis, and schizophrenia were identified. Another limitation is the lack of any human study which backs up the cautionary measures in interpreting the effect of honey. Further studies are required to address these challenges and provide detailed mechanistic insight into the use of this liquid gold against neurodegeneration.

## Figures and Tables

**Table 1:** Quantity (µg/kg) of Different Polyphenols in Various Types of Honey.

Active Ingredient	Honey Type							Quantification Method
	Manuka [15,16,17]	Acacia [18,19]	Eucalyptus [20]	Chestnut [21]	Cedar [22]	Sunflower [23]	Clover [24]	
Quercetin	43	200	105	46	-	200	200	HPLC
Myricetin	70	-	780	138	-	-	-	UHPLC
Galangin	35	550	-	149	-	220	-	UPLC
Apigenin	40	290	140	100	470	90	260	HPLC
Naringenin	-	60	-	350	107	169	368	RP-HPLC
Chrysin	30	130	190	680	170	-	470	UHPLC
Luteolin	38	140	66	80	464	320	550	HPLC-DAD
Pinocembrin	180	640	142	-	-	440	-	HPLC
Pinobanksin	290	148	319	-	-	-	-	HPLC
Isorhamnetin	40	90	-	-	-	-	-	HPLC
Kaempferol	150	90	147	250	670	120	172	HPLC-UV
Gallic acid	300	101	531	610	354	160	760	HPLC-UV
Caffeic acid	50	110	900	997	255	350	772	HPLC-UV
Coumaric acid	103	170	101	786	915	110	147	HPLC-UV
Chlorogenic acid	60	40	284	552	-	360	-	HPLC-DAD
Ferulic acid	-	740	368	166	220	-	531	HPLC-PDA
Syringic acid	400	300	366	202	394	-	235	HPLC-UV
Cinnamic acid	-	50	-	450	60	-	570	HPLC-UV
Benzoic acid	51	-	870	150	-	-	-	HPLC
**Total Active** **Ingredients**	1880	3849	5309	5706	3476	2539	5035	-

**Table 2:** Effects of Different Components of Honey in Various Neurological Disorders.

Disease	Component	Effect	References
Alzheimer’s Disease	Quercetin	Improves mitochondrial activity	[89]
		Activates Nrf-2 signaling	[90]
		Deceases oxidative stress	
		Reduces oxidative stress via PON2 activity	[91]
		Reduces neuroinflammation	[92,93]
		Decreases astrogliosis	
		Prevents neurodegeneration	
		Recovers cognitive disabilities	
	Myricetin	Improves memory and cognitive function	[94]
		Prevents the formation of fibrils as well as oligomers of Aβ	[95]
		Anti-tau protein effect	
		↓ Acetylcholinesterase	[96]
		↑ Acetylcholine	
		Exhibits anti-inflammatory activity	[97,98]
		Inhibition of the NF-κB pathway and NLRP3 inflammasome	
		Prevents the formation of fibrils as well as oligomers of Aβ	[99]
	Kaempferol	Decreases lipid peroxidation and senile plaque formation	[100]
		Protects from apoptotic damage	
		Regulates concentrations of antioxidative enzymes	[101]
		↓ TNF-α and inhibits inflammation	
		Exhibits anti-apoptotic activity	[102]
		Downregulates Bcl-2-associated X protein (Bax) and cleaved caspase-9	
		Increases neuronal density in the hippocampus	[103]
		Downregulates inflammatory proteins	[104]
		Protects from oxidative damage	[105]
	Luteolin	Alleviates oxidative stress	[106]
		Scavenges free radicals	
		Downregulates inflammatory and apoptotic proteins	
		Improves memory deficits	[107]
		Maintains neuronal density	
	Galangin	Diminishes autophagy	[108]
		Decreases levels of p-tau, β-secretase, and Aβ_42_	
		↑ Acetylcholine	[109]
		Improves cognitive functions	
	Caffeic acid	↓ Acetylcholinesterase	[110]
		↓ Inflammation	
		↓ Oxidative stress	
	Cinnamic acid	Attenuates the formation of amyloid plaques	[111]
		Activates PPARα	
		Improves memory functions	
Parkinson’s Disease	Quercetin	Protects from degeneration	[112]
		↑ Dopamine levels	
		↑ Brain-Derived Neurotrophic Factor expression	
		↑ Energy production of mitochondria	
		Restores activities of antioxidant enzymes	[113]
		Repairs cognitive deficits	
		↓ Endoplasmic reticulum stress	[114]
		↓ C/EBP homologous protein (CHOP)	
		↑ Autophagy	
		↑ Beclin-1 expression	
	Myricetin	↓ Degeneration	[115]
		↑ Dopamine levels	
		Inhibits inflammation	[116]
		Prevents the activation of microglia	
		↓ Expression of inflammatory cytokines	
	Chrysin	↑ Expression of a survival factor MEF2D	[117]
		Inhibits MAO-B	
		Alleviates oxidative stress	[118]
		Prevents inflammation and dysfunction of Na+, K+-ATPase pump	
		Ameliorates neuronal loss	[119]
		Improves memory	
	Ellagic acid	Inhibits MAO activity	[120]
		Prevents the loss of neurons	
		↓ Oxidative stress	
		Modulates levels of antioxidant enzymes	[121]
		Protects from oxidative insult	
		Diminishes apoptosis	[122]
		Inhibits MAO-B	
		Modulates ERβ/Nrf2/HO-1 signaling cascade	
	Cinnamic acid	Protects neurodegeneration	[123]
		Activates Peroxisome Proliferator Activating Receptor α (PPARα)	
	Galangin	Inhibits microglial activation	[124]
		Suppresses inflammatory factors	
		Activates PPAR-γ	[125,126,127]
		Inhibits inflammation	
		Prevents apoptosis	
		Protects from oxidative damage	
	Apigenin	Exhibits antioxidative function	[128]
		Inhibits MAO	
		Prevents apoptosis	
		Inhibits caspase-3 activation	
Huntington’s Disease	Quercetin	Improves motor functions	[129]
		Regulates peroxisome proliferator-activated receptor gamma, coactivator (PGC-1), or sirtuins (SIRT1)	
		↑ Energy production of mitochondria	
		Ameliorates behavioral malfunctions	[130]
		Exhibits anxiolytic effect	
	Myricetin	Reduces aggregation of polyglutamine	[131]
		↓ Proteo toxicity	[132]
		Repairs behavioral changes	
	Chrysin	Upregulates anti-apoptotic factor	[133]
		Downregulates pro-apoptotic factor	
		Restores neurobehavioral functions	[134]
		↑ Serotonin	
	Chlorogenic acid	Protects from genotoxicity	[135]
Amyotrophic Lateral Sclerosis	Quercetin	Inhibits aggregation of Cu-Zn superoxide dismutase (SOD)	[136,137]
	Kaempferol	Prevents cell death	[138,139]
		Reduces aggregation of superoxide dismutase 1 (SOD1)	
		↑ AMPK phosphorylation	
		Inhibits mTOR phosphorylation	
		Boosts up autophagy	
	Coumaric acid	Ameliorates oxidative stress and endoplasmic reticulum stress	[140]
		↑ Autophagy	
	Gallic acid	↑ Levels of antioxidant enzymes	[141]
		↓ Lipid peroxidation	
		Downregulates inflammatory cytokines such as TNF-α, IL-6, IL-β, and NF-κB	
		Improves motor functions	
		Prevents glutamate excitotoxicity	[142]
		Inhibits the formation of neurofibrillary tangles	
		Improves motor skills	
		↓ TDP-43 proteotoxicity	[143]
		Attenuates seizures	
Epilepsy	Quercetin	Inhibits activation of microglial cells and inflammatory cytokines	[144]
		Attenuates neurodegeneration	
		Inhibits expression of the gene for GABA receptors	[145]
		Reduces depression	
		Restores tryptophan levels	[146]
		Exerts anticonvulsant effect	
		Modulates Glycinergic and GABAergic ion channels	[147]
		Reduces behavioral signs of seizures	
		↓ Neuronal loss	[148]
		↓ Astrocyte activation	
	Myricetin	Ameliorates intensity of seizures	[149,150]
		Inhibits apoptosis	
		Downregulates Bad, Bax, and cleaved caspase 3	
		Upregulates Bcl-2 and Bcl-xL	
		Normalizes glutamate/GABA	
	Luteolin	Improves cognitive deficits	[151]
		↑ Expression of Brain-Derived Neurotrophic Factor (BDNF)	
		Exhibits anticonvulsant effect	[152]
		↑ Activation of receptors for GABA_A_	
		Reduces oxidative stress	
		Improves cognition impairments	[153]
		Modulates CaM-CaMPK signaling pathway	
		Attenuates seizures	
	Chrysin	Modulates GABA_A_ receptors	[154]
		Abrogates convulsion-induced oxidative damage	
		↓ The severity of epileptic seizures	[155]
		↓ Apoptosis	
		Boosts the expression of Nrf2, NQO-1, and HO-1	
	Apigenin	Reduces neuronal loss	[156]
		↓ Release of cytochrome c from mitochondria	
		Alleviates apoptosis	
		Inhibits overexpression of hypochlorite (HClO)	[157,158]
		↓ Oxidative damage	
		Averts cognitive impairments	[159]
		Exerts antidepressant and anti-anxiolytic effects	
		↑ Expression of Brain-Derived Neurotrophic Factor (BDNF)	
	Ferulic acid	Palliates depression	[160]
		↓ Levels of proinflammatory cytokines such as TNF-α and IL-1β	
		Inhibits Cyclooxygenase 2 (COX2) activity	
		Modulates corticosterone levels	
		Ameliorates oxidative stress	[161]
		Upregulates neuroprotective Heat shock protein 70 (Hsp70) and neurotransmitters such as Serotonin (5-HT) and norepinephrine	
		Diminishes oxidative stress	[162]
		Repairs cognitive deficits and seizure activity	
		Improves memory functions and learning capacities	[163]
		Inhibits apoptotic process	
		Scavenges free radicles	
	Naringenin	↑ Antioxidant enzymes	[164]
		Restores neuronal morphology	
		↓ Neurodegeneration	
		Impedes occurrence of seizures	[165]
		↓ Granule cell disruption (GCD) in the hippocampus	
		Ameliorates generation of proinflammatory cytokines	
		Exerts anticonvulsant effect	[166]
		Agonist effect on GABAA receptors	
		↓ Glutamate transmission	
Schizophrenia	Quercetin	Scavenges free radicals	[167]
		↑ Levels of antioxidant enzymes	
		Reduces depressive behaviors	[168]
		Improves behavioral impairments	[169]
		Boosts up antipsychotic therapy	[170]
Depression	Quercetin	↑ Levels of antioxidant enzymes	[171,172]
		↓ Decrease levels of inflammatory cytokines	
		Accrues serotonin level	
		Mitigates depressive behaviors	[173]
		Modulates levels of BDNF	
	Myricetin	Recovers hopeless behaviors	[174,175]
		Regulates BDNF levels	
		Exerts antioxidant effect	
	Kaempferol	Exert anti-depressive effects	[176]
		↑ Levels of antioxidant enzymes	
		Upregulates AKT/β-catenin cascade	
	Chrysin	Upregulates nerve growth factor (NGF) and Brain-Derived Neurotrophic Factor (BDNF)	[177]
		Normalizes Na^+^, K^+^-ATPase activity	
		Employs anti-depressant effects	[178]
		Inhibits kynurenine pathway	
		Elevates the levels of serotonin (5HT)	
	Naringenin	Performs anti-depressive activity	[179]
		Restores antioxidant enzymes’ levels	
		Modulates serotonin levels	
		Downregulates inflammation mediators	
		Inhibits acetylcholinesterase activity	[180]
		Mitigates oxidative damage	
		Upregulates BDNF, Sonic Hedgehog (Shh) signaling, NKX2.2, and PAX6	[181]
	Coumaric acid	Improves behavioral hopelessness	[182]
		Lessens inflammation-associated alterations	
		Enhances neurotrophic activity	
	Ferulic acid	Abrogates depression-like behaviors	[183]
		Downregulates pro-inflammatory cytokines	
		Downregulates factors associated with inflammation and apoptosis	[184]
		Mitigates depression	

## Data Availability

Not applicable.
